# 

**Published:** 1898-01-08

**Authors:** 


					r
3.
'Be HOSPITAL. ABSOLUTELY PURE, therefore BEST. Jan. 8th, 1898.
CADBURY'S ^ff ^ CADBURY'S
COCOA inr^ Irf /JE^ COGOA
SbK?i?!Srslpnrityatpresent % mm <4|gu. mo alkalies used.
WHICH HOSPI TAL
0, 58^ Yol. XXIII. Saturday,
t K?. 58
Jan. 8ih, 1898.
[SnbscriptionTenu^] pRICE TWOPENCE.

				

